# Induced Pluripotent Stem Cells Generated from *P0-Cre;Z/EG* Transgenic Mice

**DOI:** 10.1371/journal.pone.0138620

**Published:** 2015-09-18

**Authors:** Yasuhiro Ogawa, Akira Eto, Chisato Miyake, Nana Tsuchida, Haruka Miyake, Yasuhiro Takaku, Hiroaki Hagiwara, Kazuhiko Oishi

**Affiliations:** Department of Pharmacology, Meiji Pharmaceutical University, 2-522-1 Noshio, Kiyose, Tokyo, 204–8588, Japan; Instituto Butantan, BRAZIL

## Abstract

Neural crest (NC) cells are a migratory, multipotent cell population that arises at the neural plate border, and migrate from the dorsal neural tube to their target tissues, where they differentiate into various cell types. Abnormal development of NC cells can result in severe congenital birth defects. Because only a limited number of cells can be obtained from an embryo, mechanistic studies are difficult to perform with directly isolated NC cells. Protein zero (P0) is expressed by migrating NC cells during the early embryonic period. In the *P0-Cre;Z/EG* transgenic mouse, transient activation of the P0 promoter induces Cre-mediated recombination, indelibly tagging NC-derived cells with enhanced green fluorescent protein (EGFP). Induced pluripotent stem cell (iPSC) technology offers new opportunities for both mechanistic studies and development of stem cell-based therapies. Here, we report the generation of iPSCs from the *P0-Cre;Z/EG* mouse. *P0-Cre;Z/EG* mouse-derived iPSCs (P/G-iPSCs) exhibited pluripotent stem cell properties. In lineage-directed differentiation studies, P/G-iPSCs were efficiently differentiated along the neural lineage while expressing EGFP. These results suggest that P/G-iPSCs are useful to study NC development and NC-associated diseases.

## Introduction

Neural crest (NC) cells are a migratory, multipotent cell population that arises at the neural plate border. After delamination from the roof plate, multipotent NC cells migrate from the dorsal neural tube to their target tissues. During the migration process, NC cells retain a characteristic phenotype. However, upon reaching their target tissue, they differentiate into a wide range of cell types, including neurons and glial cells of the sensory, autonomic and enteric nervous systems, melanocytes, endocrine cells, smooth muscle cells of the heart and great vessels, and skeletal muscle and bone [1].

Recently, the fate of these unique migratory, multipotential cells has been studied using NC-specific Cre recombinase and *lacZ* or green fluorescent protein (GFP) reporter mice to facilitate genetic marking of the NC in mice. Transgenic lines that carry Cre recombinase in a NC-specific manner include protein zero (P0), Wnt1, Pax3, and HtPA [2–7]. The genetic-fate mapping revealed that the migratory NC is a collection of heterogeneous progenitors including various types of intermediate precursors and highly multipotent cells [8].

P0 is a major protein component of myelin in the peripheral nervous system, which is expressed by a subset of migrating NC cells, but not before detaching from the neuroepithelium during the early embryonic period. No other markers are specifically expressed in NC cells after emigration from the neural tube in mammals. Therefore, the P0 promoter-driven Cre-*loxP* DNA recombination system can be applied as a NC cell lineage marker [2]. In the *P0-Cre;Z/EG* double-transgenic mouse, transient activation of the P0 promoter induces Cre-mediated recombination, indelibly tagging NC-derived cells with enhanced GFP (EGFP) expression.

Recent advances in somatic cell reprogramming to induced pluripotent stem cells (iPSCs) has allowed the generation of patient-specific cells for regenerative medicine and disease modeling [9]. iPSC-derived NC cells are a valuable tool for modeling aspects of NC development, including cell fate specification, multipotency, and migration. Despite this progress, the NC cells generated by currently existing methods are highly heterogeneous populations [10–13], and it is unclear whether these iPSC-derived NC cells contain all of heterogeneous NC subpopulations. Therefore, it is critical to identify reliable markers to understand the signaling pathways necessary and sufficient for NC induction of iPSCs. In the present study, we generated iPSCs from the *P0-Cre;Z/EG* double-transgenic mouse (P/G-iPSCs), which provides a basis for mechanistic studies of NC development as well as a potential model to study NC-associated diseases.

## Materials and Methods

### Generation of *P0-Cre;Z/EG* transgenic mice

All animal procedures were performed in accordance with the Guidelines for Animal Experimentation of the Japanese Association for Laboratory Animal Science, and approved by the Institutional Animal Use and Care Committee of Meiji Pharmaceutical University. *P0-Cre* transgenic mice (P0Cre-A; provided by Dr. Marco Giovannini, INSERM, France [14] through the RIKEN BioResource Center (RBRC01459)) were maintained by breeding homozygous mice. *Z/EG* (*lacZ*/EGFP) double-reporter mice (The Jackson Laboratories, Bar Harbor, ME) that express EGFP after removal of β-geo under the control of the *CAG* promoter (chicken β-actin promoter with cytomegalovirus enhancer) were maintained by breeding heterozygous mice. *P0-Cre;Z/EG* double-transgenic mice were generated by crossing *P0-Cre* mice with *Z/EG* mice, and the F1 generation were used in this study. Mice from F1 litters were genotyped by PCR to confirm presence of the transgenes using DNA from embryo. Genotyping of *Cre* was performed using the primer pair; Cre-1 (5′- AATGCTTCTGTCCGTTTGCC-3′) and Cre-2 (5′-CTACACCAGAGACGGAAATC-3′), yielding a product of 562 bp. Genotyping of Z/EG was performed using the primer pair; Z/EG-1 (5′-ATGGTGAGCAAGGGCGAGGA-3′) and Z/EG-2 (5′-CTGCTTGTCGGCCATGATATAGACG-3′), yielding a product of 474 bp. For embryonic staging, the morning of vaginal plug was designated as embryonic day 0.5 (E0.5).

### GFP staining of *P0-Cre;Z/EG* embryos

Whole embryos and small and large intestines were stained with 4’, 6-diamidino-2-phenylindole (DAPI) and an anti-GFP antibody. For frozen cross-sections of the trunk, intestines, and brains, the tissues were fixed in 4% paraformaldehyde followed by cryoprotection in 30% sucrose in phosphate buffer and then embedded in Tissue-Tec OCT compound. The frozen cross-sections were cut using a cryostat and subjected to immunohistochemical analysis. Fluorescence images were obtained under a confocal laser-scanning microscope (FluoView 500; Olympus, Tokyo, Japan) or fluorescence microscope (Axio Imager. M2, Carl Zeiss, Jena, Germany).

### Differentiation of primary spheres prepared from *P0-Cre;Z/EG* embryonic intestines at E12.5

The small and large intestines (E12.5) were dissected out and dissociated by pipetting after incubation at 37°C for 3 min in a trypsin solution (1.33 mg/ml trypsin, 0.67 mg/ml hyaluronidase, and 0.2 mg/ml kynurenic acid in Hank’s balanced salt solution). Viable cells were plated at 5×10^5^ cells/ml in N2 medium containing 20 ng/ml basic fibroblast growth factor (bFGF) and 2 μg/ml heparin, and maintained at 37°C with 5% CO_2_. The number of primary spheres generated was assessed at 2–3 days after plating. Spheres were collected by centrifugation for 5 min at 450×g, dissociated mechanically to single cell suspensions, and replated onto poly-L-ornithine/fibronectin-coated glass coverslips. The cells were then cultured for 7 days in Dulbecco’s modified Eagle’s medium (DMEM) supplemented with 10% fetal bovine serum (FBS). Then, the differentiated cells were subjected to immunohistochemical analyses using anti-Sox10, anti-PGP9.5, anti-α-smooth muscle actin, and anti-GFP antibodies.

### Generation of iPSCs

Neural stem cells (NSCs) and primary astrocytes were prepared from *P0-Cre;Z/EG* mouse embryos on E12.5 as described elsewhere [15]. Transgenes encoding Klf4, Oct3/4, Sox2, and c-Myc were introduced into NSCs from *P0-Cre;Z/EG* mice by EBNA1/oriP-based episomal vectors as described previously [15]. On day 8–12 post-transfection, colonies with a morphology similar to that of wild-type (WT) iPSC colonies were picked up for expansion on mouse embryonic fibroblast (MEF) feeder cells in DME-iPS medium (DMEM supplemented with 15% FBS, 2 mM L-glutamine, 0.1 mM nonessential amino acids, 0.1 mM 2-mercaptoethanol) containing 1000 U/ml leukemia inhibitory factor (LIF; ESGRO). Alternatively, to generate iPSCs, primary astrocytes cultured from the *P0-Cre;Z/EG* transgenic mouse cortex were infected with lentiviral vectors carrying reprogramming transgenes according to the method of Lin *et al*. [16].

### Teratoma formation

Teratoma formation was performed as described elsewhere [15]. Briefly, approximately 5×10^5^ cells suspended in 100 μl of a Matrigel-GFR/medium mixture were injected subcutaneously into the dorsal flanks of nude mice (Balb/c slc−nu/nu, Foxn1−/−; Nihon SLC, Shizuoka, Japan). Mice were sacrificed at 3–5 weeks post-injection, and the injection sites were dissected, fixed with 4% paraformaldehyde in phosphate buffer, and then embedded in Tissue-Tec OCT. Subsequently, 25 μm-thick sections were analyzed by hematoxylin-eosin staining and immunostaining.

### Differentiation of P/G-iPSCs through the formation of spherical aggregates

Undifferentiated P/G-iPSCs (clone 61) cultured on feeder cells were dissociated to single cells in 0.25% trypsin-EDTA and then quickly reaggregated in DME-iPS medium (3000 cells/150 μl/well) using 96-well low cell adhesion plates (Sumilon Spheroid Plates, Sumitomo Bakelite Co., Tokyo, Japan) to initiate embryoid body (EB) formation. After 7 days, cell aggregates in suspension cultures were plated onto gelatin-coated dishes in differentiation medium (DMEM supplemented with 10% FBS, 4 mM L-glutamine, 0.1 mM nonessential amino acid, and 0.1 mM 2-mercaptoethanol). After 10 days, the cells were stained with antibodies against cell type-specific markers. For neural priming, undifferentiated P/G-iPSCs cultured on feeder cells were trypsinized, plated onto tissue culture dishes, and incubated for 30 min at 37°C to allow feeder cells to attach. Unattached P/G-iPSCs were collected, dissociated with 0.25% trypsin-EDTA to form a single-cell suspension, and then plated onto gelatin-coated tissue culture dishes and cultured in DME-iPS medium supplemented with 3 μM of the glycogen synthase kinase-3β (GSK-3β) inhibitor CHIR99021 and 1 μM of the MEK inhibitor PD0325901. After 2 days of culture, undifferentiated P/G-iPSCs under feeder-free conditions were cultured for 2–3 days in N2/B27 medium (DMEM/F12 plus N2/B27 supplements) in the presence or absence of 1 μM retinoic acid (RA) and/or 1 mM valproic acid (VPA). The cells were dissociated to single cells in 0.25% trypsin-EDTA. The cells were then quickly reaggregated (3000 cells/150 μl/well) using 96-well low cell-adhesion plates in Glasgow’s modification of Eagles medium (GMEM) supplemented with 10% knockout serum replacement (KSR) in the presence of 10 μM CHIR99021. The aggregates were incubated for 10 days at 37°C with 5% CO_2_. For quantitative measurement of positive cells, aggregates were dissociated into single-cell suspensions using 0.25% trypsin-EDTA to facilitate quantitative measurement of positive cells. Suspension samples were plated onto poly-L-ornithine-coated glass coverslips and incubated for 30 min to allow adhesion. For each sample, 20 fluorescence images of different fields (1.3 × 1.8 mm) were obtained using a fluorescence microscope (Axio Imager. M2, Carl Zeiss, Jena, Germany). The percentages of GFP-positive cells (% of total DAPI count) were then determined using ImageJ (National Institutes of Health, Bethesda, MD). Values represent the mean ± S.E. from five independent experiments.

### Immunostaining and alkaline phosphatase staining

Immunostaining and alkaline phosphatase (AP) staining were performed as described previously [15]. Primary antibodies were anti-stage-specific embryonic antigen 1 (SSEA1, MC-480; Developmental Studies Hybridoma Bank, DSHB, Iowa City, IA), anti-Oct3/4 (MBL, Nagoya, Japan), anti-nestin (Rat-401; DSHB), anti-α-smooth muscle actin (Progen, Heidelberg, Germany), anti-neuronal class III β-tubulin (Tuj1; Covance, Richmond, CA), anti-Sox2 (Millipore, Bedford, MA), anti-glial fibrillary acidic protein (GFAP; Dako, Carpenteria, CA), anti-α-actinin (Sigma-Aldrich, Tokyo, Japan), anti-α-fetoprotein (R&D Systems, Minneapolis, MN), anti-Sox1 (Cell Signaling Tech, Beverly, MA), anti-Sox10 (Millipore), anti-PGP9.5 (Ultra Clone, Wellow, Isle of Wight, UK), and anti-GFP (Nakarai Tesque, Kyoto, Japan). Secondary antibodies were Alexa Fluor 568-conjugated goat anti-mouse IgG, Alexa Fluor 488-conjugated goat anti-mouse IgG1, Alexa Fluor 568-conjugated goat anti-mouse IgM, Alexa Fluor 647-conjugated goat anti-mouse IgG, Alexa Fluor 647-conjugated goat anti-rat IgG, and Alexa Fluor 488-conjugated goat anti-rabbit IgG (all purchased from Molecular Probes, Life Technologies, Eugene, OR).

### RT-PCR

RT-PCR was performed as described previously [15].

### Reagents

CHIR99021 and BIO were purchased from Tocris (Ellisville, MO). PD0325901, SB431542, LDN193189, VPA, DMEM, DMEM/F12, GMEM, and N2 supplement were purchased from Wako Co. (Tokyo, Japan). RA, heparin, and DAPI were obtained from Sigma-Aldrich (Tokyo, Japan). Tissue-Tec OCT compound was purchased from Sakura Finetek USA Inc. (Torrance, CA). bFGF was from R&D Systems (Minneapolis, MN). LIF (ESGRO) was from Chemicon International Inc. (Temecula, CA). Matrigel-GFR was from BD Biosciences (San Jose, CA). KSR, B27 supplement, and TO-PRO-3 were from GIBCO/BRL Life Technologies (Grand Island, NY). All other chemicals were reagent grade.

### Statistical analysis

Values are expressed as the means ± standard error (S.E.). The significance of differences between experimental groups was analyzed using the unpaired Student's *t*-test.

## Results

### EGFP-positive NC-derived cells in *P0-Cre;Z/EG* transgenic mouse embryos and primary cultured cells

**Fig 1:**
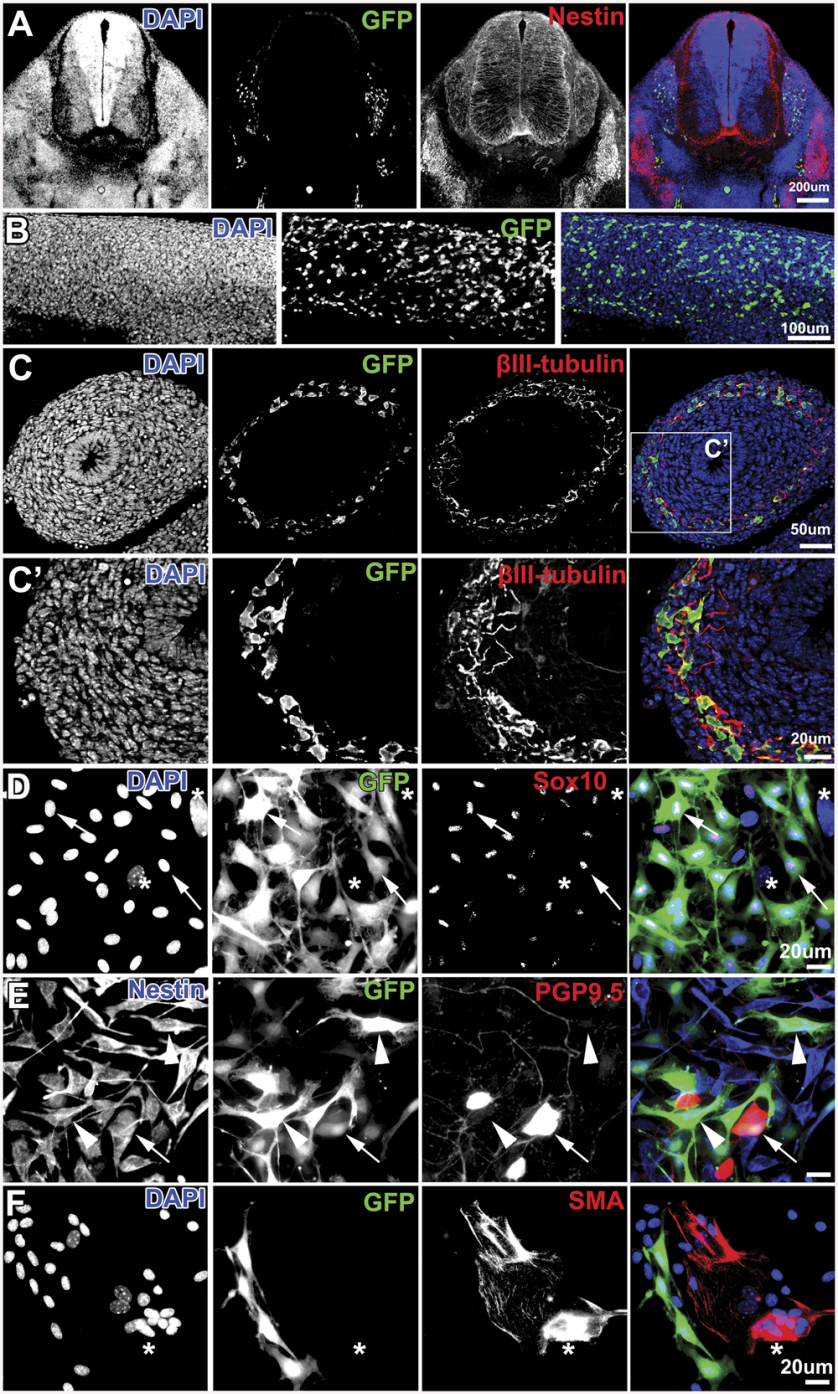
GFP-positive cells are NC-derived cells. A, Immunostaining of a transverse section through the *P0-Cre;Z/EG* embryonic trunk at E12.5 for GFP (green) and nestin (Red). Blue represents DAPI staining. Scale bar indicates 200 μm. B, Whole mount GFP staining of *P0-Cre;Z/EG* embryonic intestines at E12.5 (green). Blue represents DAPI staining. Scale bar indicates 100 μm. C and C’, Immunostaining of cross sections through the intestine (E12.5) for GFP (green) and βIII-tubulin (red). Blue represents DAPI staining. Scale bars indicate 50 μm (C) and 20 μm (C’). D, Immunostaining of differentiated cells from intestine-derived primary spheres for GFP (green) and Sox10 (Red). Arrows indicate Sox10 and GFP double-positive cells. Stars indicate double-negative cells. Blue represents DAPI staining. E, Immunostaining of differentiated cells from intestine-derived primary spheres for Nestin (blue), GFP (green), and PGP9.5 (red). Arrows indicate Nestin/PGP9.5/GFP triple-positive cells. Arrowheads indicate Nestin/GFP double-positive cells. F, Immunostaining of differentiated cells from intestine-derived primary spheres for GFP (green) and α-smooth muscle actin (SMA, red). Stars indicate single GFP-positive cells. Blue represents DAPI staining. Scale bars indicate 20 μm (C–F).

Crossing *P0-Cre* transgenic mice with *Z/EG* (*lacZ/EGFP*) double-reporter mice enabled us to trace NC-derived cells by *P0* promoter-driven EGFP expression. GFP-positive cells were detected in E9.5 embryos (S1A Fig), indicating that the P0 promoter is already active at the early stages of NC cell differentiation. These cells were found in E12.5 spinal dorsal root ganglia and the enteric nervous system, in which NC-derived cells have been reported to be present [2] (Fig 1A–1C). In E11.5, E12.5, E14.5, E17.5, P0, and adult intestines, a subpopulation of p75 neurotrophin receptor (p75, marker of NC stem cells)-positive cells were GFP-positive (S1 Fig). This observation indicated that, although the P0 promoter is not necessarily activated in all neural crest derivatives, P0 is expressed in a subpopulation of NC cells. In addition, GFP-positive cells were positive for Sox10 (marker of early migrating NC cells) in E14.5 and P0 intestines (S1G and S1H Fig), βIII-tubulin (neuronal marker) in E12.5 intestines (Fig 1C), PGP9.5 (neuronal marker) in P0 and adult intestines (S2A and S2B Fig), GFAP (marker of astrocytes and Schwann cells) in P0 and adult intestines (S2C and S2D Fig), and α-smooth muscle actin (smooth muscle cell marker) in the P2 brain (S2E Fig). To evaluate the self-renewal and differentiation capabilities of NC-derived cells, dissociated intestinal cells from E12.5 embryos were induced to form neurosphere-like spheres that were cultured under differentiation conditions. Immunostaining revealed that some GFP-positive cells were also positive for Sox10, nestin (neural precursor marker), and PGP9.5 (Fig 1D and 1E), indicating that NC cells are present in E12.5 intestines and able to differentiate toward the neural lineage. On the other hand, we did not observe double-positive cells for GFP and α-smooth muscle actin (Fig 1F).

### Generation of iPSCs from *P0-Cre;Z/EG* mice

Transgenes encoding Klf4, Oct3/4, Sox2, and c-Myc were introduced into NSCs from *P0-Cre;Z/EG* transgenic mice by EBNA1/oriP-based episomal vectors as described previously [15, 17]. At 4 weeks after transfection, colonies were picked up based on their morphological resemblance to mouse WT-iPSC colonies. Three clones (#61, 64, and 71) propagated robustly as colonies when maintained on MEFs. Alternatively, to generate iPSCs, primary astrocytes cultured from the *P0-Cre;Z/EG* transgenic mouse cortex were infected with lentiviral vectors carrying reprogramming transgenes according to the method of Lin *et al*. [16]. As a result, we obtained two clones (#7 and 23). The episomally derived clone #61 was used for further evaluation in this study.

**Fig 2:**
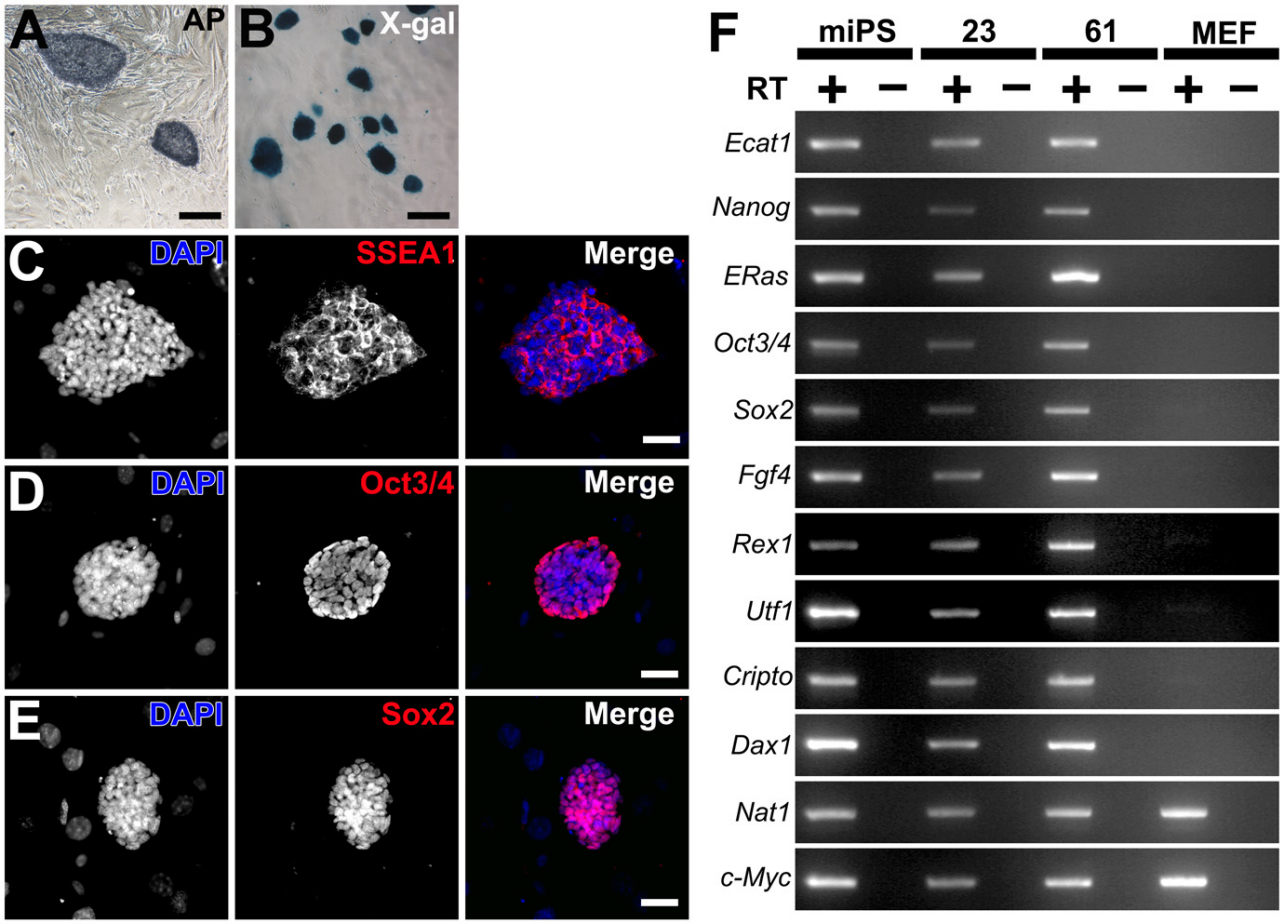
Characterization of iPSCs derived from NSCs of *P0-Cre;Z/EG* mice (P/G-iPSCs). AP staining (A), X-gal staining (B), and immunostaining (C; SSEA-1, D; Oct3/4, and E; Sox2) of P/G-iPSCs (clone 61) grown on MEF feeder cells. Scale bars indicate 200 μm (A, B) and 100 μm (C–E). F, RT-PCR analysis of ESC marker gene expression in WT-iPSCs, P/G-iPSCs (clones 23 and 61), and MEFs. Nat1 was used as an internal control. PCR products were amplified from cDNA samples with (+) or without (−) reverse transcriptase.

To determine whether the clones expressed pluripotency markers, we verified the presence of AP activity and immunostained for embryonic stem cell (ESC) markers, SSEA1, Oct3/4, and Sox2. As a result, the colonies were positive for both AP activity and the ESC markers (Fig 2A, 2C, 2D and 2E). These colonies were stained with X-Gal, indicating no P0 promoter-derived Cre-loxP recombination (Fig 2B). RT-PCR analysis showed that the putative iPSC clones 23 and 61 from *P0-Cre;Z/EG* transgenic mice (P/G-iPSCs) were positive for 10 ESC marker genes including Ecat1, Nanog, ERas, Oct3/4, Sox2, Fgf4, Rex1, Utf1, Cripto, and Dax1 (Fig 2F). The expression of these genes in the P/G-iPSC clones was similar to that in mouse WT-iPSCs, but absent in MEFs. As described previously, c-Myc is expressed in MEFs, ESCs, and WT-iPSCs [18].

**Fig 3:**
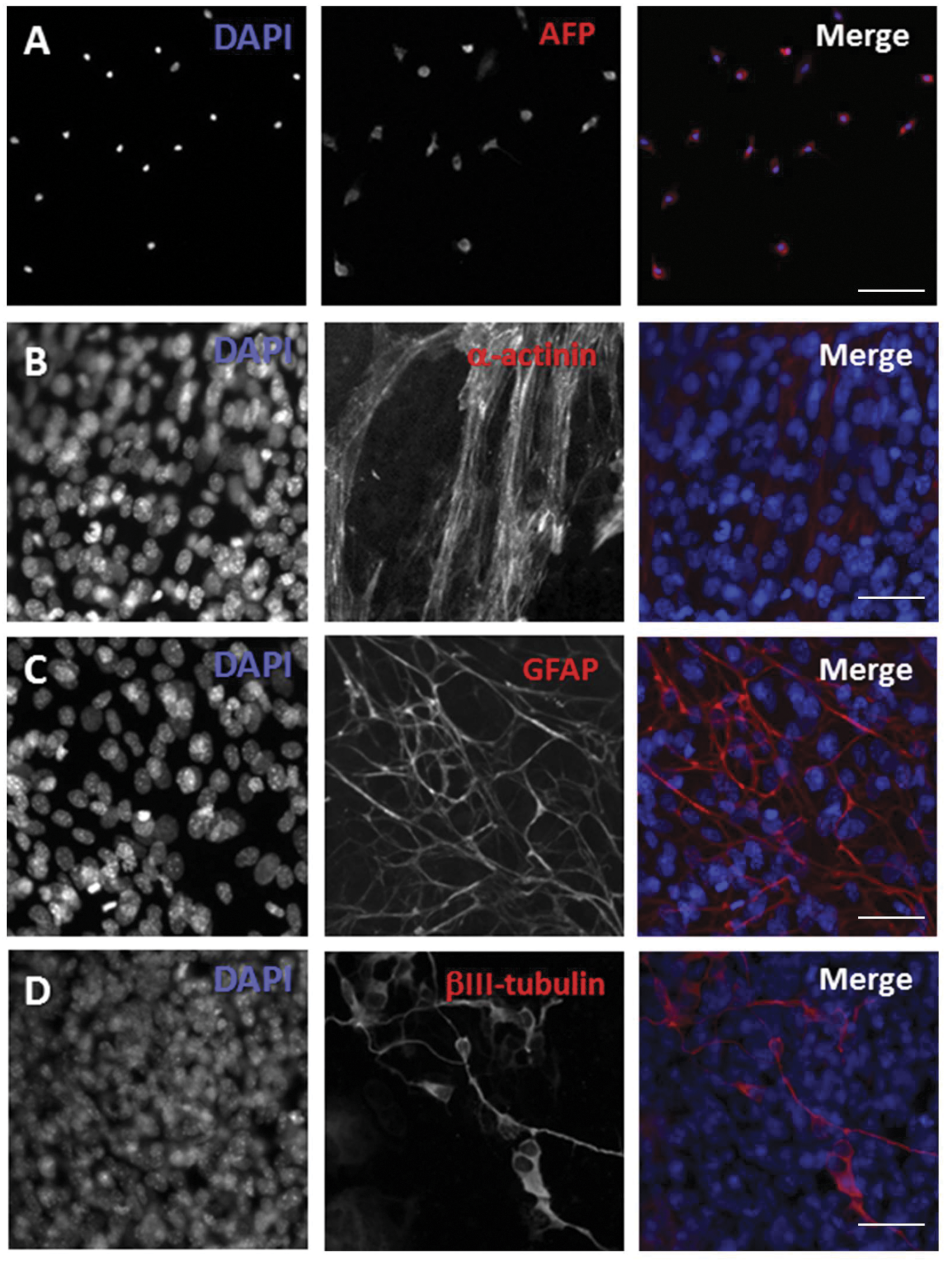
Differentiation of P/G-iPSCs into cell types of all three germ layers. Immunostaining showing that α-fetoprotein (A), α-actinin (B), GFAP (C), and βIII tubulin (D) were expressed in spontaneously differentiated P/G-iPSCs. Blue represents DAPI staining. Scale bars indicate 50 μm.

**Fig 4:**
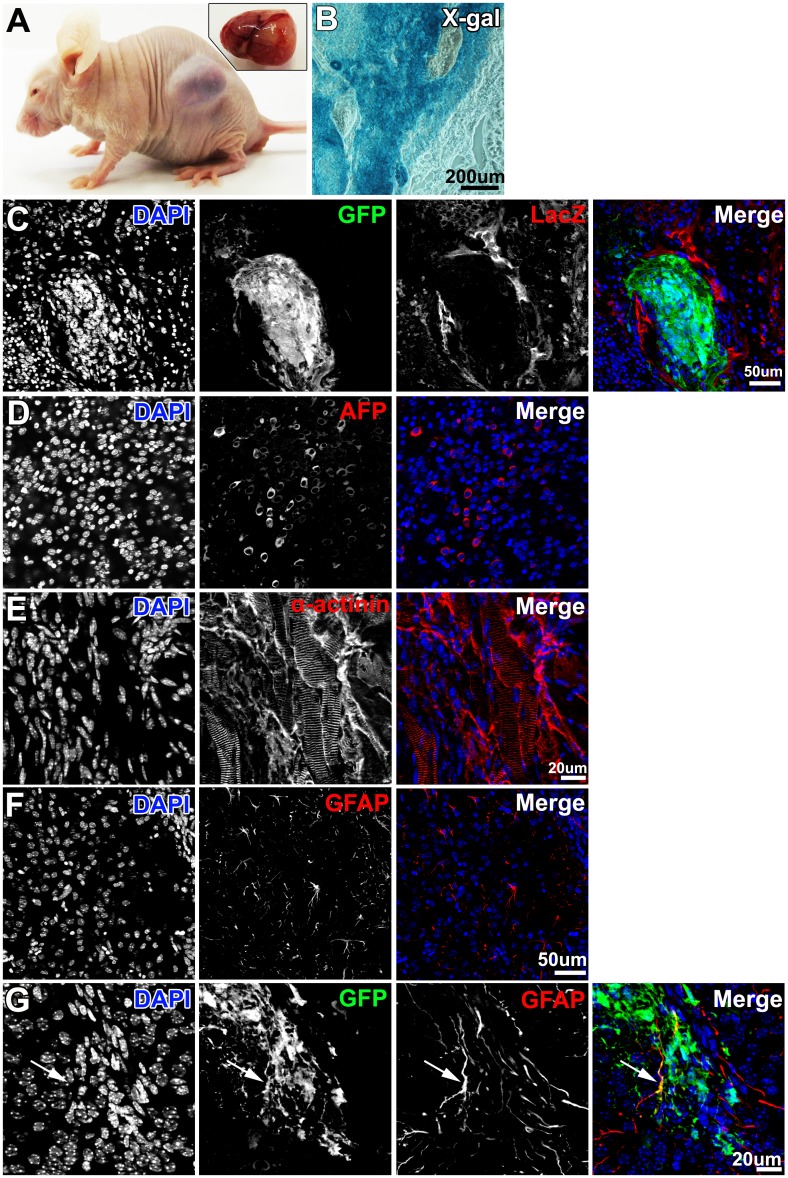
Teratoma formation of P/G-iPSCs. A, Teratoma formation of P/G-iPSCs (clone 61) after transplantation into a nude mouse. The inset shows a removed tumor. B, X-gal staining of tumors. C, Immunostaining of tumors for GFP (green) and LacZ (red). D–F, Immunostaining of tumors for α-fetoprotein (D, red), α-actinin (E, red), and GFAP (F, red). G, Immunostaining of tumors for GFP (green) and GFAP (red). Arrows indicate GFP and GFAP double-positive cells. Blue represents DAPI staining. Scale bars indicate 200 μm (B), 50 μm (C, D, F), and 20 μm (E, G).

To determine whether P/G-iPSCs (clone #61) differentiated into cell types of various germ layers, the P/G-iPSCs were allowed to differentiate spontaneously in EB cultures. Formation of EBs was achieved by culturing P/G-iPSCs in DME-iPS medium on low-attachment plates for 7 days, followed by transfer to gelatin-coated dishes for further culture. After 10 days, immunostaining showed that the cells were positive for α-fetoprotein (endoderm marker), α-actinin (mesoderm marker), GFAP (ectoderm marker), and βIII tubulin (ectoderm marker) (Fig 3). Next, we verified the pluripotency of P/G-iPSCs in teratoma formation assays. After injection of P/G-iPSCs into immunocompromised mice, P/G-iPSCs formed teratomas containing tissue derivatives of the endoderm, mesoderm, and ectoderm (Fig 4). We concluded that P/G-iPSCs can spontaneously differentiate into derivatives of all three germ layers.

### Differentiation of P/G-iPSCs into NC-derived cells

**Fig 5:**
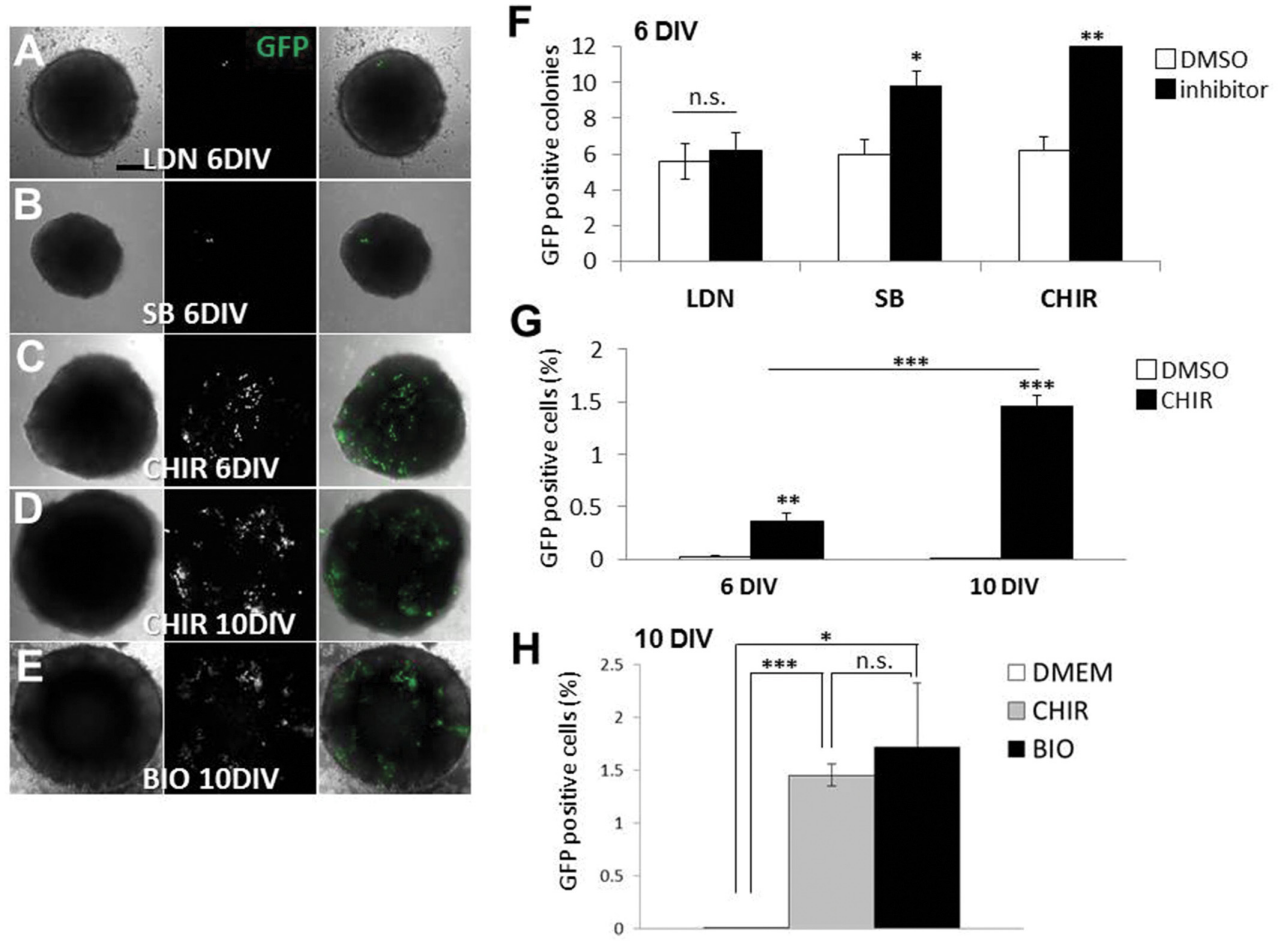
Effects of small molecule inhibitors on the differentiation of P/G-iPSCs into GFP-positive cells. A–E, P/G-iPSCs (clone 61) were dissociated to single cells in 0.25% trypsin-EDTA and quickly reaggregated (3000 cells/150 μl/well) using 96-well low-adhesion plates in GMEM supplemented with 10% KSR in the presence of 0.01 μM LDN193189 (A), 10 μM SB431542 (B), 10 μM CHIR99021 (C, D), or 1 μM BIO (E). The aggregates were incubated for up to 6 (A–C) or 10 (D, E) days. Scale bars indicate 200 μm. F, GFP-positive aggregates were counted in 12 wells. Values represent the mean ± S.E. from five independent experiments. G, After 6 or 10 days of culture, the aggregates were dissociated to single cells in 0.25% trypsin-EDTA. The single cell suspensions were plated onto poly-L-ornithine-coated coverslips and incubated for 30 min to allow adhesion. The percentages of GFP-positive cells were then determined. Values represent the mean ± S.E. from five independent experiments. H, Comparison of GSK-3β inhibitors CHIR99021 and BIO for differentiation of P/G-iPSCs into GFP-positive cells. After 10 days of culture in the presence of 10 μM CHIR99021 or 1 μM BIO, the percentages of GFP-positive cells were determined. Values represent the mean ± S.E. from five independent experiments. ***P<0.005. **P<0.01. *P<0.05. n.s.: Difference not significant (*P*>0.05). LDN, LDN193189. SB, SB431542. CHIR, CHIR99021. DIV, days *in vitro*.

Regulation of specific developmental pathways, including bone morphogenetic protein (BMP)/activin and Wnt signaling axes, is required for proper NC development during embryogenesis [19]. We first used specific inhibitors for these pathways to investigate their effects on differentiation of P/G-iPSCs (clone #61) into NC-derived cells through the formation of spherical aggregates in serum-free GMEM. CHIR99021 effectively increased the numbers of GFP-positive aggregates (Fig 5C, 5D and 5F). After 6 or 10 days in culture, the cell aggregates were dissociated to single cells to determine the percentages of GFP-positive cells. We found that the percentages of GFP-positive cells were 0.36 ± 0.08% and 1.46 ± 0.11% (mean ± S.E., n = 5) after 6 or 10 days in culture, respectively (Fig 5G). SB435142, a small molecule inhibitor of the activin type-I receptor ALK4 and nodal type-I receptor ALK7, also increased the percentages of GFP-positive cells in aggregates but to a lesser extent compared with CHIR99021 (Fig 5B and 5F). However, LDN193189 [20], a potent, synthetic small molecule inhibitor of BMP type-I receptors ALK2 and ALK3, had no effect (Fig 5A and 5F). BIO, another inhibitor of GSK-3β, also increased the percentages of GFP-positive cells to the same extent as CHIR99021 (Fig 5E and 5H).

### Neural priming with RA followed by GSK-3β inhibition promotes NC differentiation

**Fig 6:**
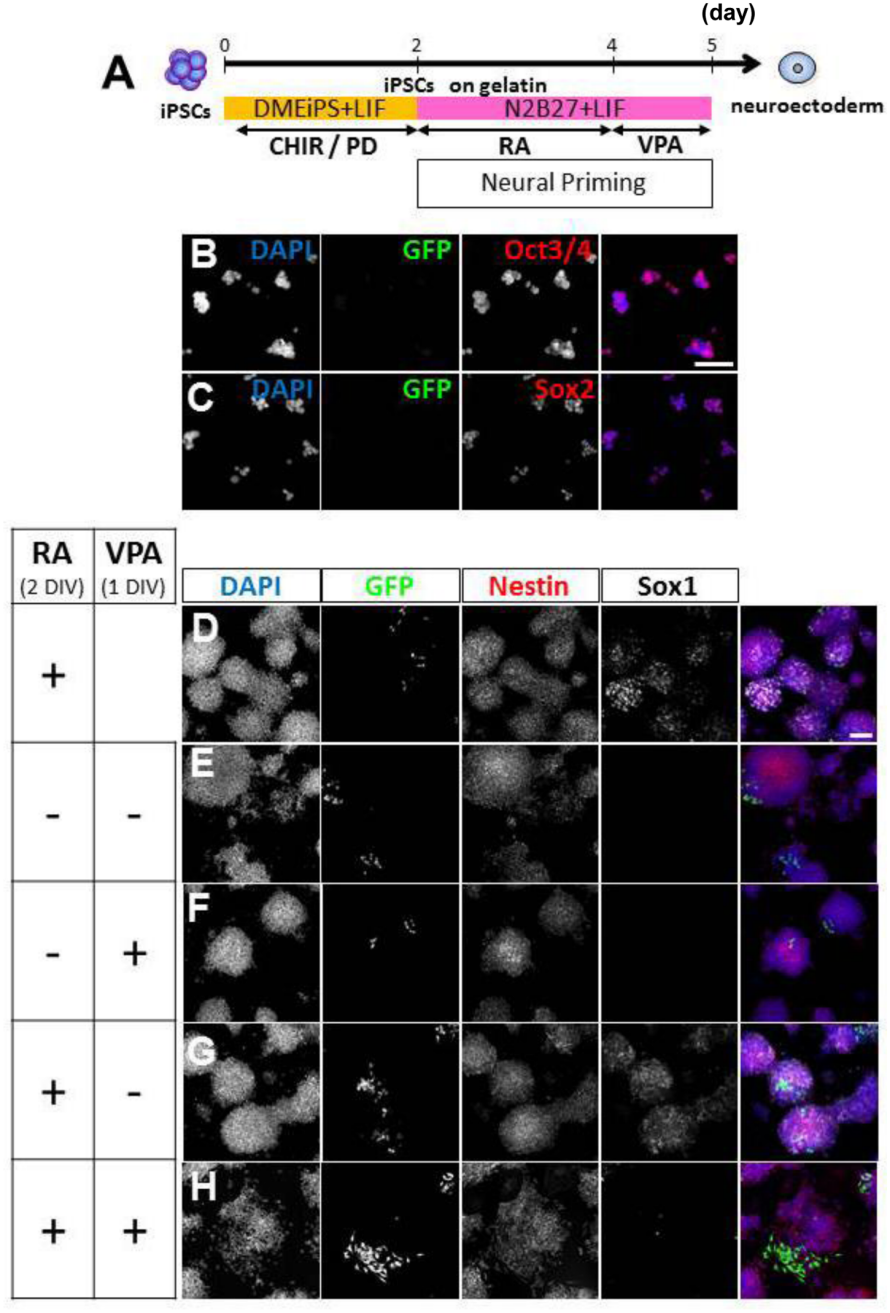
Culture of P/G-iPSCs under feeder-free conditions and RA supplementation in N2/B27 medium promote neural induction. A, Schematic diagram of the culture procedure for neural priming. Undifferentiated P/G-iPSCs on feeder cells were trypsinized, plated onto tissue culture dishes, and incubated for 30 min at 37°C to allow feeder cells to attach. Unattached P/G-iPSCs were collected, dissociated into single cells in 0.25% trypsin-EDTA, and then plated onto gelatin-coated tissue culture dishes and cultured in DME-iPS medium with 3 μM CHIR99021 and 1 μM PD0325901 (B, C). After 2 days of culture, undifferentiated P/G-iPSCs under feeder-free conditions were cultured for 2 (D) or 3 (E–H) days in N2/B27 medium in the presence or absence of 1 μM RA and/or 1 mM VPA. B–H, Immunostaining for GFP (green), Oct3/4 (red), Sox2 (red), Nestin (red), and Sox1 (white). Blue represents DAPI staining. Scale bars indicate 100 μm. CHIR, CHIR99021. PD, PD0325901. RA, retinoic acid. VPA, valproic acid. DIV, days *in vitro*.

**Fig 7:**
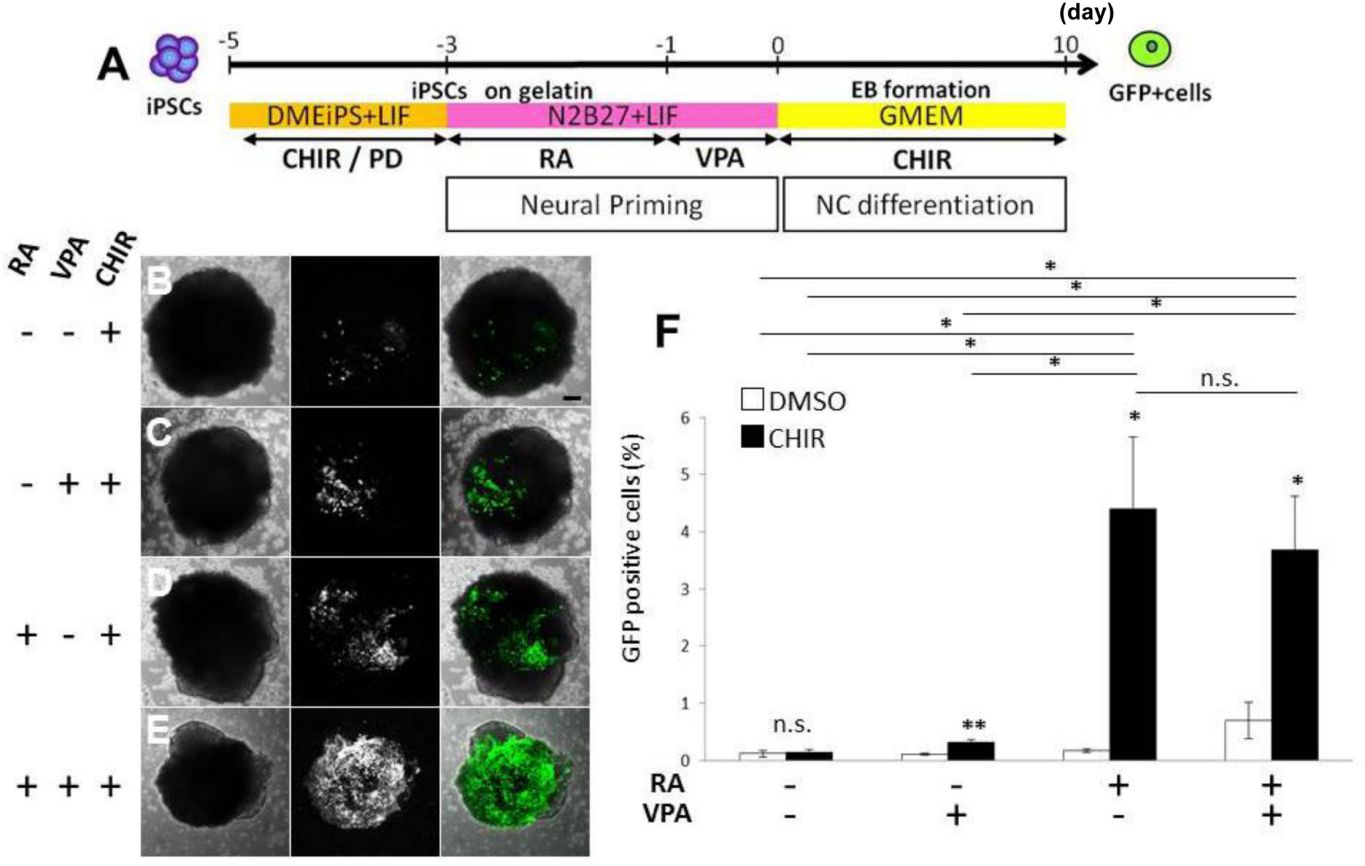
Neural priming and CHIR99021 treatment efficiently induce differentiation of P/G-iPSCs into GFP-positive cells. A, Schematic diagram of the culture procedure for differentiation. Undifferentiated P/G-iPSCs cultured for 3 days in N2/B27 medium in the presence or absence of 1 μM RA and/or 1 mM VPA were dissociated into single cells in 0.25% trypsin-EDTA and quickly reaggregated (3000 cells/150 μl/well) using 96-well low-adhesion plates in GMEM supplemented with 10% KSR in the presence of 10 μM CHIR99021. The aggregates were incubated for 10 days at 37°C with 5% CO_2_. B–E, Representative image of GFP-expression. Scale bars indicate 100 μm. F, After 10 days of culture with CHIR99021, the percentages of GFP-positive cells were determined. **P<0.01. *P<0.05. n.s.: Difference not significant (*P*>0.05). Values represent the mean ± S.E. from five independent experiments. CHIR, CHIR99021. PD, PD0325901. RA, retinoic acid. VPA, valproic acid.

To develop a more effective method to generate NC cell derivatives from P/G-iPSCs (clone #61), we first cultured P/G-iPSCs on gelatin under feeder-free conditions [21] and primed them with N2/B27 medium to promote neural induction [13]. P/G-iPSCs cultured on gelatin under feeder-free conditions in the presence of PD0325901 and CHIR99021 were positive for ESC markers including Oct3/4 and Sox2, but negative for GFP signals (Fig 6B and 6C). Cells primed with N2/B27 medium for 3 days were positive for nestin, indicating efficient promotion of neural induction (Fig 6E). The percentages of nestin-positive cells in N2/B27 and DME-iPS medium were 91.7 ± 3.3% and 74.6 ± 8.0%, respectively (mean ± S.E., n = 5, *P*<0.001). RA treatment for 2 days did not change the percentages of nestin-positive cells (92.1 ± 3.6%). GFP-positive cells were observed in some colonies (Fig 6D–6H), although no significant increase in the number of GFP-positive cells was observed when the cells were treated with RA for the first 2 days. In addition, expression of the neuroectodermal marker Sox1 was significantly promoted in the presence of RA (Fig 6D and 6G). These neural primed cells were then subjected to NC lineage induction through the formation of spherical aggregates in serum-free GMEM containing CHIR99021 (Fig 7A). After 10 days of culture, the aggregates were dissociated to single cells to determine the percentages of GFP-positive cells. We found that the percentages of GFP-positive cells were increased significantly by RA treatment at the neural priming stage. The percentages of GFP-positive cells in the presence or absence of RA were 4.39 ± 1.24% and 0.14 ± 0.03% (mean ± S.E., n = 5), respectively (Fig 7B, 7D and 7F). Additional treatment with VPA, a histone deacetylase inhibitor, at the neural priming stage did not affect the percentages of GFP-positive cells (Fig 7D–7F). These results suggest that neural priming with RA followed by GSK-3β inhibition with CHIR99021 efficiently promotes the differentiation of P/G-iPSCs into NC-derived cells.

### Multipotentiality of P/G-iPSC-derived GFP-positive cells

**Fig 8:**
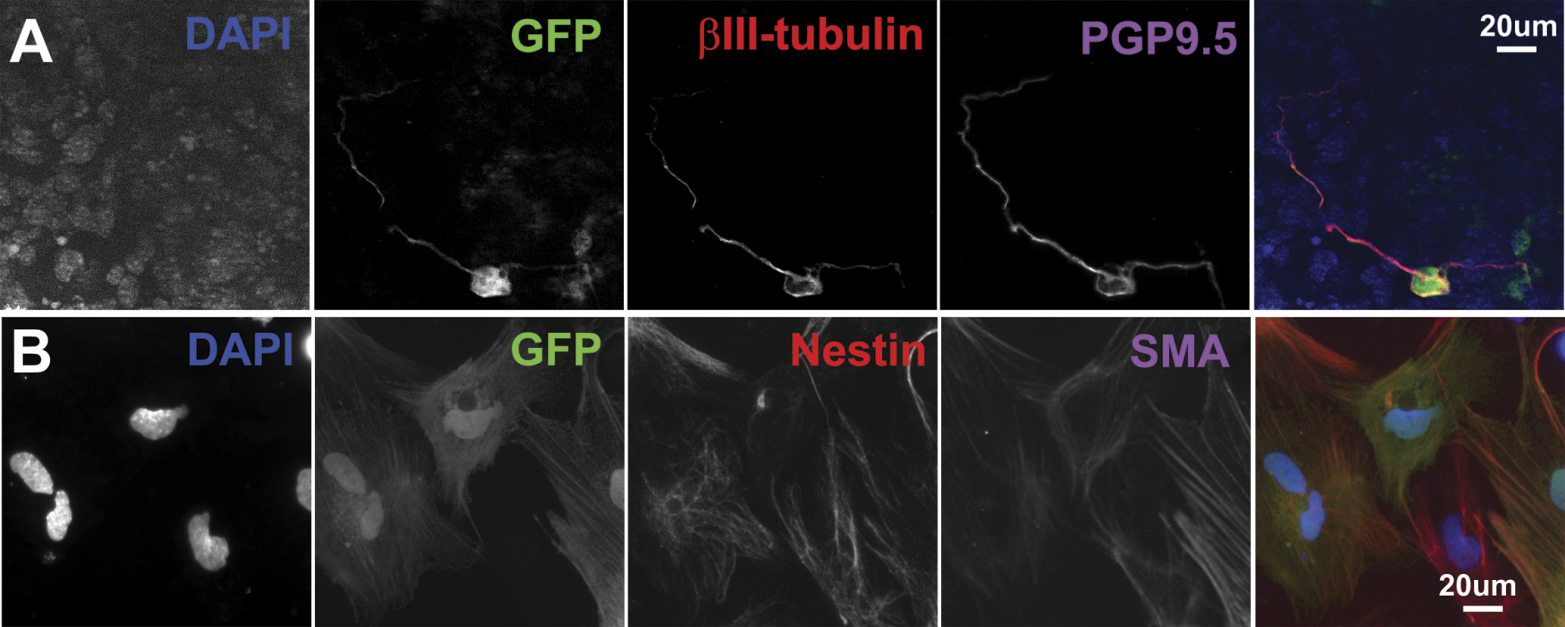
Multipotentiality of P/G-iPSC-derived GFP-positive cells. Neural primed cells were induced to form spherical aggregates in the presence of CHIR99021. After 4 days of culture, the aggregates were cultured further to allow adhesion onto fibronectin-coated glass coverslips for 7 days in N2/B27 medium in the presence of CHIR99021. A, Immunostaining for GFP (green), βIII-tubulin (red), and PGP9.5 (purple). In B, the aggregates were dissociated into single cells, quickly reaggregated and cultured in N2 medium (DMEM/F12 plus N2 supplement) containing 20 ng/ml bFGF and 2 μg/ml heparin for 3 days to form neurospheres, and then cultured under differentiation conditions. Immunostaining for GFP (green), nestin (red), and α-smooth muscle actin (SMA, purple). Blue represents DAPI staining. Scale bars indicate 20 μm.

To determine the differentiation capabilities of GFP-positive cells derived from P/G-iPSCs (clone #61), neural primed cells were induced to form spherical aggregates in the presence of CHIR99021. After 4 days of culture, the aggregates were cultured further to allow adhesion onto fibronectin-coated glass coverslips for 7 days in N2/B27 medium in the presence of CHIR99021. Immunostaining showed that GFP-positive cells were positive for βIII-tubulin and PGP9.5 (Fig 8A). The aggregates were also dissociated into single cells, quickly reaggregated, and cultured in N2 medium in the presence of bFGF for 3 days to form neurospheres, and then cultured under differentiation conditions. GFP-positive cells were also positive for nestin and α-smooth muscle actin (Fig 8B). These results suggest that GFP-positive cells derived from P/G-iPSCs exhibit a multipotent differentiation potential.

## Discussion

In the present study, we established iPSCs from a *P0-Cre;Z/EG* double-transgenic mouse, which provides a new system to study NC development. The P/G-iPSCs described here exhibit pluripotent stem cell properties based on their similarities to murine iPSCs in terms of gene expression, proliferation, and the ability to differentiate spontaneously into cells of different germ layers. Moreover, we have differentiated these cells into NC cells and their derivatives that can be marked by EGFP. These iPSCs provide an advantage to investigate differentiation of iPSCs toward the neural lineage.

Most of the early fate-mapping analyses were performed using avian or amphibian chimeric embryos, with more recent studies using retroviral labeling in chick embryos [22]. Fukiishi and Morriss-Kay were the first to use vital dye methods to follow cranial NC cell migration in mammals [23]. In recent years, promoter-reporter constructs [24] and double-transgenic analysis [25] using *lacZ* or GFP reporter mice and NC-specific Cre recombinase have facilitated genetic marking of NC in mice. The *P0-Cre* mouse line [2] that expresses Cre in an NC-specific manner is well characterized and widely used to study post-migratory NC cells [7, 26–31]. In the present study, we showed that GFP-positive cells were found in the spinal dorsal root ganglia and enteric nervous system, in which NC-derived cells has been reported to be present [2]. Therefore, we used *P0-Cre;Z/EG* double-transgenic mice to generate iPSCs.

Understanding and triggering the signaling pathways necessary and sufficient for NC induction in iPSCs were critical goals in this study. The protocol can also be applied to patient-derived iPSCs, and thus used to further our knowledge of human diseases associated with NC development, including Hirschprung’s disease. It is known that the regulation of specific developmental pathways, including BMP/activin and Wnt signaling axes, are required for proper NC development during embryogenesis [19]. Efficient methods to generate neural progenitor cells from human pluripotent stem cells, including human iPSCs and ESCs, have been recently developed by applying specific inhibitors. Such inhibitors include SB431542 that inhibits activin type-I receptor ALK4 and nodal type-I receptor ALK7 and LDN193189 that inhibits BMP type-I receptors ALK2 and ALK3 [20, 32, 33]. More recently, Menendez *et al*. reported that the inhibition of Activin A/Nodal signaling and concurrent activation of Wnt signaling can also induce NC cell formation from pluripotent stem cells [34, 35]. In the present study, CHIR99021, a small molecule that inhibits GSK-3β and activates Wnt signaling, effectively increased the numbers of GFP-positive aggregates. BIO, another inhibitor of GSK-3β, also increased the percentages of GFP-positive cells to the same extent as CHIR99021. These results suggest that Wnt signaling pathways are required for NC differentiation in the EB formation method.

It is well known that the neural precursor stage is necessary for NC induction [13]. At the neural priming stage, we treated P/G-iPSCs with RA in N2/B27 medium. RA is one of the most important morphogens, and its embryonic distribution correlates with neural differentiation and positional specification in the developing nervous system. For this reason, RA is thought to be one of the most important extrinsic inductive signals for neural differentiation of pluripotent stem cells *in vitro* [36]. To develop a more effective method to generate NC cell derivatives from P/G-iPSCs, we primed them with N2/B27 medium to promote neural induction [13]. Sox1 is the earliest known specific marker of neuroectoderm in the mouse embryo [37], which is expressed in the neural plate of mouse embryos at E7.5 [38]. After delamination from the roof plate, Sox1-positive cells migrate from the dorsal neural tube to their target tissues where they differentiate into neurons and glial cells of sensory, autonomic and enteric nervous systems. RA promotes the expression of Sox1 in pluripotent stem cells [39]. The priming in the presence of RA significantly increased the expression of both Sox1 and nestin, indicating that priming with RA efficiently promotes differentiation into neuroectodermal cells. When neural primed cells were then subjected to NC lineage induction through the formation of spherical aggregates in the presence of CHIR99021, there was a significant increase in the percentages of GFP-positive cells, indicating that RA is necessary for the induction of NC cells [40]. These results suggest that Sox1-positive neuroectodermal cells induced by priming with RA alone can easily differentiate into GFP-positive NC cells when Wnt is subsequently activated by CHIR99021.

Histone deacetylases (HDACs) are transcriptional regulators that deacetylate histones to modify the chromatin structure [41, 42] or deacetylate transcription factors to modulate their activity [43]. Because histone deacetylation leads to local compaction of the chromatin structure, HDACs are generally regarded as transcriptional repressors. However, HDACs are also needed to maintain genes in an active state. Jacob *et al*. reported that HDAC1/2, essential transcriptional regulators of NC specification into Schwann cells and satellite glia, activate P0 transcription [44]. Therefore, the level of histone acetylation and the presence of HDACs at a gene locus do not always reflect the activity status of a gene. Hence, we investigated the possible involvement of HDACs at the neural priming stage. One day of treatment with VPA, a HDAC inhibitor, followed by RA treatment during neural priming decreased the expression of Sox1, indicating that Sox1 transcription activated by RA is downregulated by inhibition of HDAC. However, additional treatment with VPA at the neural priming stage did not influence the percentages of GFP-positive cells compared with those obtained by priming with RA alone. These results suggest that the first 2 days of treatment with RA at the priming stage is sufficient for the subsequent induction of GFP-positive NC cells by Wnt activation, and that sustained expression of Sox1 at day 3 in the neural priming stage is not necessarily required.

In this study, neural priming with RA induced Sox1-positive neuroectodermal cells, and concomitant GSK-3β inhibition with CHIR99021 efficiently promoted differentiation into NC-derived cells. Wnt signaling is implicated in the regulation of cell growth and differentiation during central nervous system development [45, 46]. It remains to be clarified whether activation of Wnt signaling selectively promotes the differentiation of Sox1-positive neuroectodermal cells into the NC lineage. This study provides a basis for the generation of patient-specific NC cells to study disease-modeling aspects of NC development, including cell fate specification, multipotency, and migration. Our data also demonstrate that the generation of NC cells and their derivatives might be useful in cell replacement therapy for the treatment of neurocristopathies, including Hirschsprung’s disease.

In conclusion, we generated iPSCs from a *P0-Cre;Z/EG* mouse, which exhibit pluripotent stem cell properties. In lineage-directed differentiation studies, P/G-iPSCs were efficiently differentiated along the neural lineage while maintaining EGFP expression. These results suggest that P/G-iPSCs are useful to study NC development and disease.

## Supporting Information

S1 Fig Fluorescence images of *P0-Cre;Z/EG* transgenic mouse embryos. Frozen cross-sections were immunostained as described in the Materials and Methods. Fluorescence images were obtained under a confocal laser-scanning microscope (FluoView 500; Olympus, Tokyo, Japan). A, Whole mount fluorescence images of *P0-Cre;Z/EG* transgenic mouse embryos showing GFP expression at E9.5. Arrowheads indicate GFP-positive cells. Scale bar indicates 200 μm. B, Immunostaining of a transverse section through the *P0-Cre;Z/EG* embryonic trunk at E12.5 for GFP (green) and p75 (red). Blue represents TO-PRO-3 staining. Arrows indicate GFP/p75 double-positive cells. Arrowheads indicate GFP-positive cells. Scale bar indicates 200 μm. nt, neural tube; nc, notochord; li, liver. C–F, Immunostaining of cross sections through E12.5 (C), E17.5 (D), P0 (E), and adult (F, 8 weeks of age) intestines for GFP (green) and p75 (red). Blue represents TO-PRO-3 staining. Scale bars indicate 50 μm (C, D) and 25 μm (E, F). Arrows indicate GFP/p75 double-positive cells. Arrowheads indicate GFP-positive cells. Stars indicate single p75-positive cells. G and H, Immunostaining of cross-sections through E14.5 (G) and P0 (H) intestines for GFP (green), p75 (red), and Sox10 (blue). Scale bars indicate 50 μm (G) and 25 μm (H). Crosses indicate GFP/p75/Sox10 triple-positive cells. Arrows indicate GFP/p75 double-positive cells. Arrowheads indicate GFP-positive cells. Stars indicate single p75/Sox10 double-positive cells.
(TIF)

S2 Fig Immunostaining of *P0-Cre;Z/EG* transgenic mouse sections. Frozen cross-sections were immunostained as described in the Materials and Methods. Fluorescence images of cross-sections through P0 (A, C), and adult (B, D) intestines and the P2 brain (E) were obtained under a confocal laser-scanning microscope or fluorescence microscope. A and B, GFP (green) and PGP9.5 (red). Blue represents TO-PRO-3 staining. Arrows indicate GFP/PGP9.5 double-positive cells. C and D, GFP (green) and GFAP (red). Blue represents TO-PRO-3 staining. Arrows indicate GFP/GFAP double-positive cells. E, GFP (green) and α-smooth muscle actin (SMA, red). Blue represents DAPI staining. Arrows indicate GFP/SMA double-positive cells. Scale bars indicate 25 μm.
(TIF)
