# Supplementary figures and images for: Induced Pluripotent Stem Cells Generated from P0-Cre;Z/EG Transgenic Mice
Source: PLoS One. 2015 Sep 18;10(9):e0138620. doi: 10.1371/journal.pone.0138620 (PMC4575135; doi:10.1371/journal.pone.0138620)

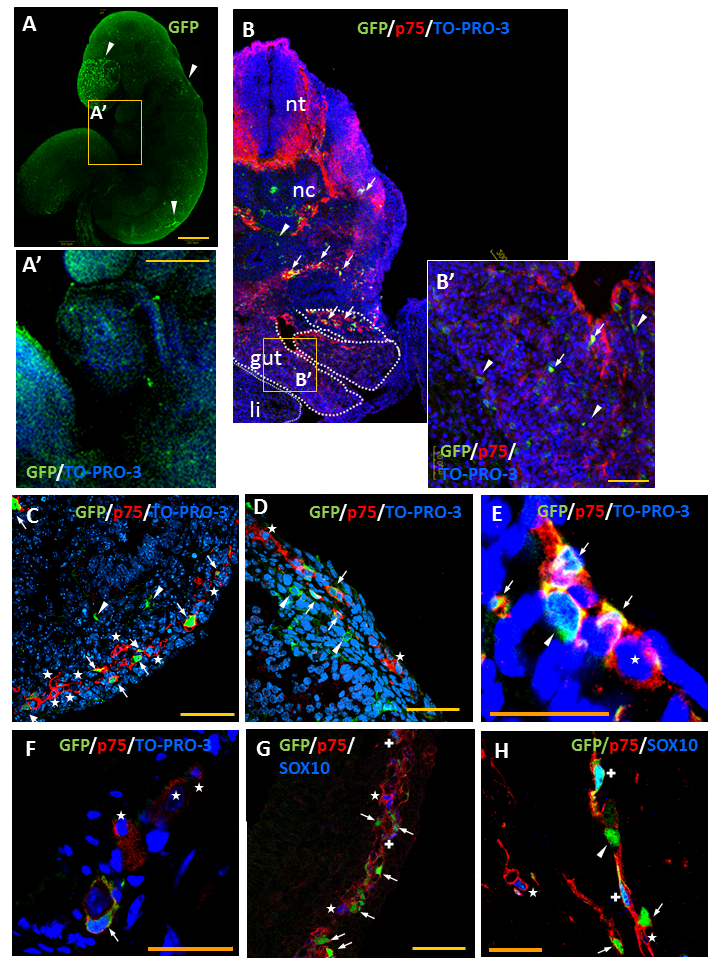

Supplement: S1 Fig — Frozen cross-sections were immunostained as described in the Materials and Methods. Fluorescence images were obtained under a confocal laser-scanning microscope (FluoView 500; Olympus, Tokyo, Japan). A, Whole mount fluorescence images of P0-Cre;Z/EG transgenic mouse embryos showing GFP expression at E9.5. Arrowheads indicate GFP-positive cells. Scale bar indicates 200 μm. B, Immunostaining of a transverse section through the P0-Cre;Z/EG embryonic trunk at E12.5 for GFP (green) and p75 (red). Blue represents TO-PRO-3 staining. Arrows indicate GFP/p75 double-positive cells. Arrowheads indicate GFP-positive cells. Scale bar indicates 200 μm. nt, neural tube; nc, notochord; li, liver. C–F, Immunostaining of cross sections through E12.5 (C), E17.5 (D), P0 (E), and adult (F, 8 weeks of age) intestines for GFP (green) and p75 (red). Blue represents TO-PRO-3 staining. Scale bars indicate 50 μm (C, D) and 25 μm (E, F). Arrows indicate GFP/p75 double-positive cells. Arrowheads indicate GFP-positive cells. Stars indicate single p75-positive cells. G and H, Immunostaining of cross-sections through E14.5 (G) and P0 (H) intestines for GFP (green), p75 (red), and Sox10 (blue). Scale bars indicate 50 μm (G) and 25 μm (H). Crosses indicate GFP/p75/Sox10 triple-positive cells. Arrows indicate GFP/p75 double-positive cells. Arrowheads indicate GFP-positive cells. Stars indicate single p75/Sox10 double-positive cells. (TIF) [file pone.0138620.s001.tif]

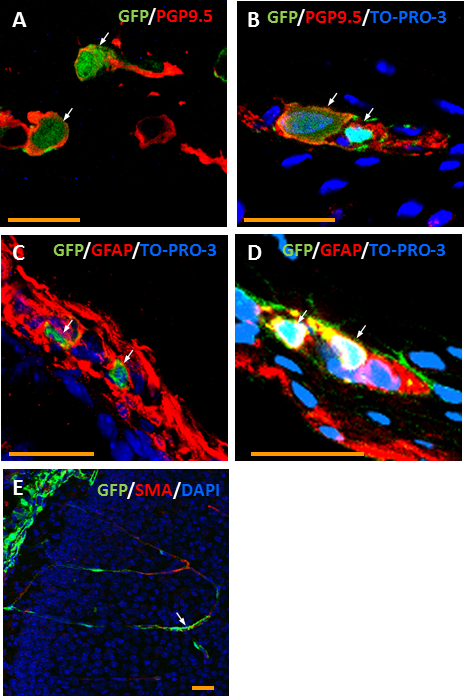

Supplement: S2 Fig — Frozen cross-sections were immunostained as described in the Materials and Methods. Fluorescence images of cross-sections through P0 (A, C), and adult (B, D) intestines and the P2 brain (E) were obtained under a confocal laser-scanning microscope or fluorescence microscope. A and B, GFP (green) and PGP9.5 (red). Blue represents TO-PRO-3 staining. Arrows indicate GFP/PGP9.5 double-positive cells. C and D, GFP (green) and GFAP (red). Blue represents TO-PRO-3 staining. Arrows indicate GFP/GFAP double-positive cells. E, GFP (green) and α-smooth muscle actin (SMA, red). Blue represents DAPI staining. Arrows indicate GFP/SMA double-positive cells. Scale bars indicate 25 μm. (TIF) [file pone.0138620.s002.tif]
